# Aerogels for Optofluidic Waveguides

**DOI:** 10.3390/mi8040098

**Published:** 2017-03-29

**Authors:** Yaprak Özbakır, Alexandr Jonas, Alper Kiraz, Can Erkey

**Affiliations:** 1 Department of Chemical and Biological Engineering, Koc University, 34450 Sarıyer, Istanbul, Turkey; yozbakir@ku.edu.tr; 2 Department of Physics, Koc University, 34450 Sarıyer, Istanbul, Turkey; 3 Department of Physics, Istanbul Technical University, 34469 Maslak, Istanbul, Turkey; 4 Department of Electrical and Electronics Engineering, Koc University, 34450 Sarıyer, Istanbul, Turkey

**Keywords:** aerogels, optofluidics, optical waveguides, microfluidics, nanostructured materials, microchannels, laser ablation

## Abstract

Aerogels—solid materials keeping their internal structure of interconnected submicron-sized pores intact upon exchanging the pore liquid with a gas—were first synthesized in 1932 by Samuel Kistler. Overall, an aerogel is a special form of a highly porous material with a very low solid density and it is composed of individual nano-sized particles or fibers that are connected to form a three-dimensional network. The unique properties of these materials, such as open pores and high surface areas, are attributed to their high porosity and irregular solid structure, which can be tuned through proper selection of the preparation conditions. Moreover, their low refractive index makes them a remarkable solid-cladding material for developing liquid-core optofluidic waveguides based on total internal reflection of light. This paper is a comprehensive review of the literature on the use of aerogels for optofluidic waveguide applications. First, an overview of different types of aerogels and their physicochemical properties is presented. Subsequently, possible techniques to fabricate channels in aerogel monoliths are discussed and methods to make the channel surfaces hydrophobic are described in detail. Studies in the literature on the characterization of light propagation in liquid-filled channels within aerogel monoliths as well as their light-guiding characteristics are discussed. Finally, possible applications of aerogel-based optofluidic waveguides are described.

## 1. Introduction

Being based on the integration of microfluidics with optics, optofluidics allows for controlling the interaction of fluids with light at small spatial scales and developing new optical devices for detection, imaging, and chemical and biological analysis [1,2,3,4]. Optofluidic waveguides that enable the controlled routing of light in integrated optofluidic systems are an important component of many of these devices.

Light guiding can be achieved by using either total internal reflection (TIR) or interference [5]. Interference-based light guides require the waveguide core region to be covered with multiple reflective layers so as to achieve near-perfect light confinement due to constructive or destructive interference. Bragg waveguides [6], Bragg fibers [7], photonic crystal fibers [8], and anti-resonant reflecting optical waveguides (ARROWs) [9,10] are primary examples of such interference-based waveguides. They require relatively complicated fabrication and coating techniques. In contrast, TIR-based waveguides can be simply obtained by a waveguide core material having a refractive index sufficiently higher than the refractive index of the surrounding cladding medium [11,12]. This principle applies to both planar waveguides and optical fibers [13]. If TIR occurs inside a waveguide, the light losses due to light leakage out of the waveguide are extremely low.

**Figure 1:**
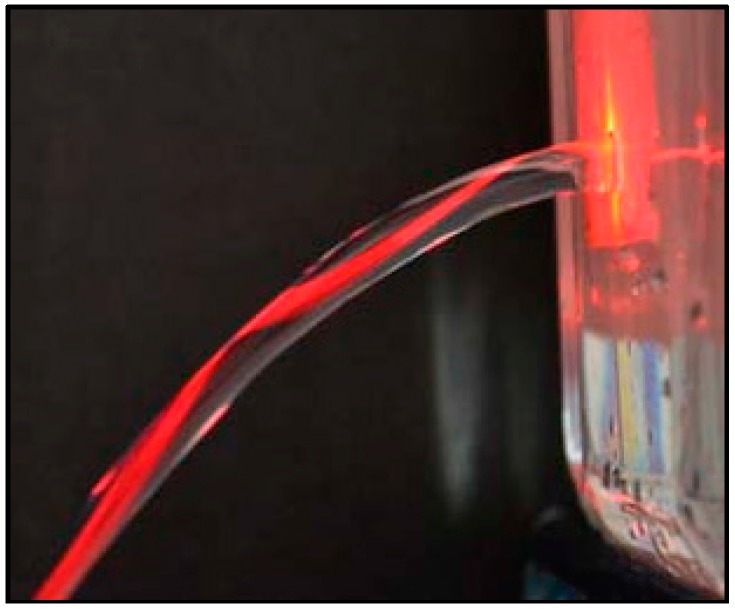
Total internal reflection (TIR) in a stream of water flowing through a hole drilled at the bottom of a glass bottle. Light from a laser source is coupled into the water stream [14]. Adapted from “Liquid Fiber Optics” by Freeberg, B., 2011 High School Physics Photo Contest, http://www.aapt.org/Programs/contests/winnersfull.cfm?id=2687&theyear=2011. Copyright [2017] by American Association of Physics Teachers. Reprinted with permission.

TIR-based liquid-core optofluidic waveguides can be easily obtained provided a low refractive index cladding medium can be found. For example, since the refractive index of water is much higher than the refractive index of the surrounding air, light can be guided in a water stream pouring out of a water-filled bottle by TIR when the incident angle of light on the surface of the water filament is greater than the critical angle (see Figure 1 [14]). However, in manufacturing optofluidic devices, a solid cladding material is typically required. In TIR-based liquid-core waveguides, the core liquid is confined within channels fabricated in a suitable solid material that simultaneously acts as the waveguide cladding [15,16]. In order to guide light by total internal reflection with low attenuation, the cladding material should be non-absorbent and have a much lower refractive index than the core material (*n*_core_ > *n*_cladding_), and a smooth interface should be present between the core and cladding material. When these requirements are met, the light remains confined in the waveguide liquid core, maintaining high intensities over long propagation distances. Devices based on TIR-based liquid core optofluidic waveguides have been attracting an increasing attention. For instance, Cai et al. [17] demonstrated direct detection and quantification of Ebola virus in an integrated optofluidic device including solid-core and liquid-core waveguides. Sample preparation and target pre-concentration were performed in a polydimethylsiloxane (PDMS)-based microfluidic chip, followed by single nucleic acid fluorescence detection in liquid-core optical waveguides on a silicon chip. Jung et al. [18] used a TIR-based liquid core optofluidic waveguide for efficient light delivery in PDMS-based photobioreactor (PBR) for cyanobacteria growth. TIR-based waveguides were built by coating a thin layer of amorphous fluoropolymer (Teflon AF) on the inner walls of PDMS channels; thus, total internal reflection occurred at the interface between the Teflon AF layer and the culture medium. They performed experiments with both regular and Teflon AF-coated PBRs for a comparison of cell growth. They demonstrated that photosynthetic cell growth improved by up to 9% in the optofluidic waveguide-based PBR compared to the regular PBR.

In applications involving aqueous solutions, the refractive index of the core liquid is around 1.33 (refractive index of water). However, a limited choice of solid host materials with an index of refraction below that of water is available; thus it is a challenge to find a cladding material with a refractive index much lower than 1.33 to maintain a reasonable numerical aperture (NA) [12,19]. Most of the polymers, including those that are utilized in lab-on-a-chip devices such as PDMS, have higher refractive indices than water; thus, light cannot be confined and delivered by TIR in channels fabricated from these polymers. Among fluoropolymers, Teflon AF has a refractive index of 1.29–1.31 that is slightly lower than the refractive index of water. Therefore, Teflon AF or a layer of Teflon AF can be used to clad a liquid-core optical waveguide. Thin coatings of amorphous Teflon AF deposited on substrates such as glass, silicone, and PDMS have been commonly employed for this purpose [20,21,22]. However, Teflon AF has poor adhesion to the common solid substrates used for manufacturing of microfluidic chips. Chemical functionalization of the surface of Teflon AF may improve adhesion, but it is very difficult to functionalize Teflon AF. Furthermore, low refractive index contrast of ~0.04 between the core liquid and Teflon AF prevents efficient propagation of light within the aqueous core [20,21].

Aerogels are nanostructured materials with an internal solid structure consisting of crosslinked polymers with a large number of air-filled pores between the solid polymeric chains. Because of their very high porosity, they are sometimes termed “solid air” and have refractive indices close to that of air [19,23,24,25,26,27]. This makes aerogels remarkable solid-cladding materials for building optofluidic waveguides with high numerical apertures without the use of coatings [19,23,25,27,28,29]. Aerogels can also be produced in any shape in appropriate molds and, therefore, they are suitable materials for the fabrication of optofluidic devices [24,30,31]. Efficient light-guiding characteristics of aerogel-based optofluidic waveguides have been demonstrated in recent studies by coupling laser light into water-filled channels fabricated in silica aerogel blocks [19,23,25,27].

This paper is a review of the literature on using aerogels for optofluidic wave-guiding applications. First, an overview of different types of aerogels and their physicochemical properties is presented. Subsequently, possible techniques for fabricating channels in aerogel monoliths are discussed and methods to make the surface of the channels hydrophobic in order to support aqueous liquid cores are described in detail. Finally, studies in the literature on the characterization of light propagation in liquid-filled channels within aerogel monoliths as well as their light-guiding characteristics are summarized. Areas that need further development in order to produce practical optofluidic devices are highlighted. Finally, possible applications of such waveguides are presented.

## 2. Aerogels

These versatile nanoporous materials can be synthesized from a wide range of molecular precursors using sol-gel chemistry, followed by removal of the solvent from the gel network using drying [31,32,33]. The unusual flexibility of the sol-gel processing enables remarkable control over the aerogel properties. Their surface properties, porosities, thicknesses, and refractive indices can be tailored to desired values by modifying the method of preparation and the composition of the reactant solution [29,30,34,35]. Thus, aerogels are attractive for a wide range of applications in areas such as aerospace, thermal insulation, and acoustic device development [32,36].

Aerogels can be generally classified into three categories: inorganic, organic, and inorganic–organic hybrids. Inorganic aerogels are synthesized using metal alkoxides as precursors and include various aerogels such as silica (SiO_2_), alumina (Al_2_O_3_), titania (TiO_2_), or zirconia (ZrO_2_) [29,30,31,37]. Silica aerogels—the most extensively studied aerogels—have pores typically ranging from 5 to 100 nm, an average pore diameter between 20 and 40 nm, porosities up to 99.8%, and bulk densities ranging from 0.003 to 0.5 g/cm^3^, which results in an extremely low refractive index in the range of 1.003–1.1 [29,36,38,39,40]. Because of their low refractive indices, they are often used in Cherenkov radiation detectors [40,41,42,43,44].

Resorcinol–formaldehyde and melamine–formaldehyde aerogels are the most commonly studied organic aerogels; they are formed from either connected colloidal particles or polymeric chains of about 10 nm in diameter. They have pores with small diameters (≤ 50 nm) and high specific surface areas (400–1000 m^2^/g) [45,46]. Carbon aerogels, which are obtained by pyrolysis of organic aerogels, are attractive, especially in energy-related applications, due to their high electrical conductivity [46].

Hybrid aerogels are synthesized by incorporation of organic polymers such as polyacrylate, polyimide, or polyurea within an inorganic aerogel matrix such as silica aerogel. Hybrid aerogels may display enhanced mechanical properties as compared with either pure organic aerogels or pure inorganic aerogels while maintaining high porosity and high surface area [34,47,48,49,50,51].

Even though aerogels were first synthesized by Samuel Kistler as early as in 1932, they remained a laboratory curiosity for a long time and were not produced on a commercial scale until 2002, primarily due to their high production costs. In 2002, Aspen Aerogels developed an aerogel-based blanket for thermal insulation by impregnating a fibrous structure with an aerogel-forming precursor solution. Subsequently, other companies such as Nano Hi-Tech also started producing aerogel blankets. Cabot Corporation (Boston, MA, USA) developed silica aerogel granules under the trade name of Nanogel^®^ with excellent insulation, water-repelling, and light transmittance properties. Nanogel^®^ is used in glazing systems such as glass, polycarbonate, acrylic, or structural composite panels due to its desirable characteristics resulting in an environmentally friendly, energy-efficient daylighting system [52,53]. Recently, BASF (Ludwigshafen, Germany) also started producing organic aerogel insulation panels under the trade name Slentite. These are mechanically strong, high-performance insulating materials with low thermal conductivity (17 mW/(m·K)) compared to other insulation materials such as mineral wool, expanded polystyrene, extruded polystyrene, and polyurethane, with thermal conductivities typically in the range of 25 to 50 mW/(m·K) [54]. The panel offers space-saving, efficient insulation, allowing the insulating layer to be 25% to 50% slimmer than conventional materials for the same insulation performance [32,52,53,54]. The above examples indicate that a wide variety of aerogels with different properties for optofluidics-related applications may be produced on a commercial scale in the future.

## 3. Aerogel-Based Optofluidic Waveguides

**Figure 2:**
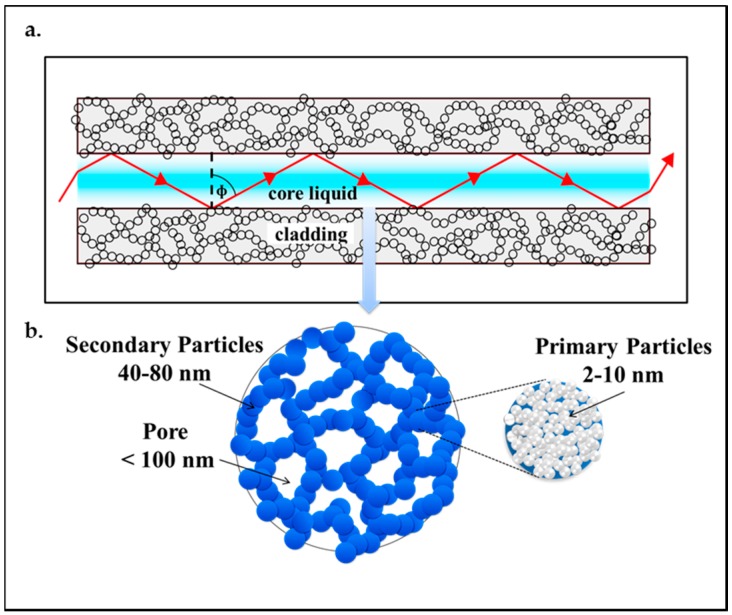
(**a**) Liquid core optofluidic waveguide created in a microchannel within a silica aerogel monolith; (**b**) nanometer-scale particles and pores in an aerogel.

Aerogel-based optofluidic waveguides are constructed by opening microchannels inside monolithic aerogel blocks. Following surface treatment, the microchannels are filled with a suitable liquid that serves as the waveguide core liquid. There is actually no restriction on the type of liquid and the type of aerogel that can be used, as long as the liquid can be confined inside the aerogel block without penetrating the aerogel network. The refractive index of any type of aerogel will almost always be less than the refractive index of any liquid. As shown in Figure 2, a microchannel in a silica aerogel is surrounded by a wall that is made up of interconnected secondary particles that are around 40 to 80 nm in size, with pockets of air in between them that constitute pores with sizes less than 100 nm. The secondary particles are formed from the primary particles, which are 2 to 10 nm in size. The air in the aerogel pockets is responsible for guiding light by total internal reflection in the liquid-filled channel [25,28,55].

**Figure 3:**
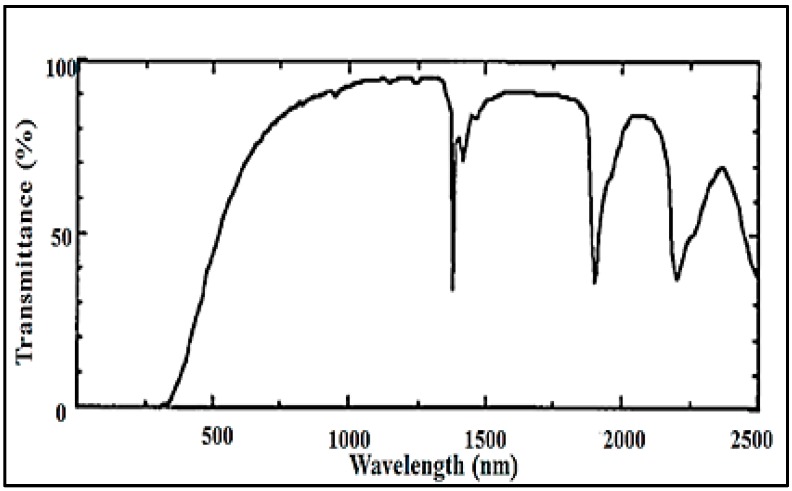
Transmission spectra of TEOS-based silica aerogel [56]. “Reproduced with permission from Tamon et al., Preparation of silica aerogel from TEOS; published by *J. Colloid Interface Sci.*, 1998.”

The losses in an aerogel-based optical waveguide are primarily due to two factors. The first is Rayleigh-type light scattering from the secondary solid particles at the interface. These losses can be calculated using the diffuse reflectance spectra. The second factor is the absorption of the optical mode propagating in the waveguide core or its evanescent tail, which penetrates into the cladding medium through the liquid core–aerogel interface. This absorption of the evanescent wave in the aerogel cladding can be related to the transmission spectra. Such a spectrum, recorded between 200 and 2500 nm for a silica aerogel sample obtained by hydrolysis tetraethylorthosilicate (TEOS) and drying by supercritical CO_2_ (scCO_2_), is given in Figure 3 [56]. The material is translucent in the visible spectral region. Since silica has no absorption band in the visible part of the spectrum, the decrease in the transmittance is predominantly due to the light scattering. In general, aerogel network contains a thin layer of adsorbed water molecules. Since the absorption of water is stronger in the near-infrared spectral region, the aerogel is less transparent in that region. In particular, the spectral absorption peaks between 900–1150 nm, 1300–1800 nm, and 1800–2000 nm can all be attributed to light absorption by adsorbed water in the aerogel network. The broad absorption band at 2200 nm then represents light absorption by the silanol group of aerogels [32,56,57].

Monolithic silica aerogels have hydroxyl groups on the surface of the secondary particles, which makes them hydrophilic. When the channel inside such a hydrophilic monolithic silica aerogel is filled with water, water penetrates the aerogel matrix surrounding the channel and the combination of water adsorption and high capillary stress due to a very small pore size leads to the erosion and collapse of the monolithic structure and, consequently, of the waveguide. Therefore, the channel walls should be rendered hydrophobic to prevent the penetration of water. This can be accomplished by replacing the hydrophilic groups on the channel walls with hydrophobic groups. Xiao et al. [25] and Eris et al. [23] showed that water can be confined in fabricated microchannels in surface-modified hydrophobic aerogels without any penetration or structure collapse. Yalızay et al. [19] demonstrated that the channels in such hydrophobic silica aerogels are also compatible with ethylene glycol. 

Another distinctive property of aerogel-based liquid core waveguides is that the open porous network of the aerogel cladding enables gases to readily diffuse out from the core liquid to the surroundings or diffuse into the core liquid from the surroundings by diffusive transport through the pores of the aerogel cladding. Moreover, air bubbles formed within the liquid in the channel can be removed, leaving a continuous, bubble-free light path. Such bubbles are not desirable as they disturb the propagation of light through the channel, leading to high optical loss from scattering and eventually causing the failure of the waveguide, which is hard to avoid in conventional channels with gas-impermeable walls.

## 4. Preparation of Aerogel Waveguides

### 4.1. Synthesis of Aerogels

**Figure 4:**
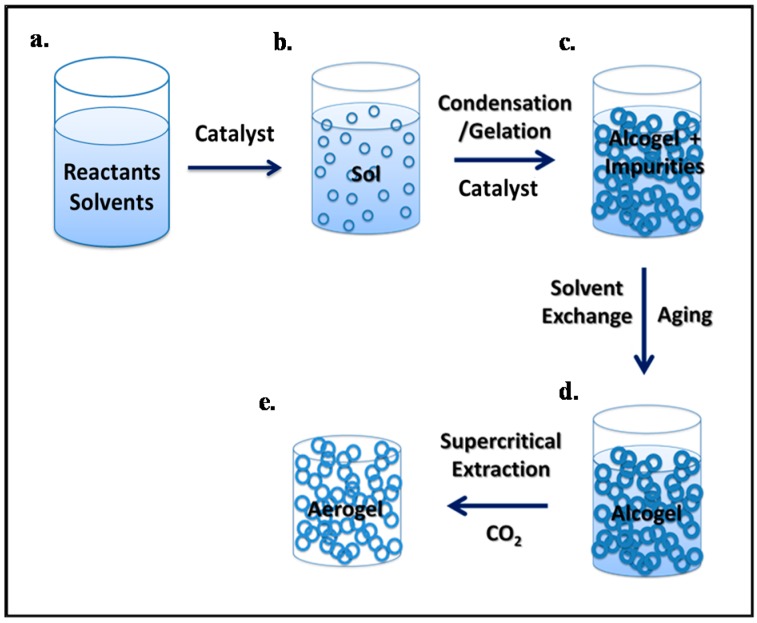
Preparation of monolithic aerogels by the sol-gel method. (**a**) Reactants and solvents are mixed in the reaction vessel; (**b**) upon addition of a catalyst, colloidal suspension (sol) is formed; (**c**) condensation of the sol leads to the formation of alcogel containing impurities; (**d**) aging process reinforces the gel skeleton structure; (**e**) supercritical drying yields the final solid dry aerogel.

Aerogels are synthesized using a conventional sol-gel process, which generally consists of the formation of a sol, the gelation of the sol solution, the aging of the gel, and the extraction of the solvent from the alcogel network, or drying of the alcogel. Typical steps used in the synthesis of aerogels are summarized in Figure 4 [29,37,58,59].

(i) Sol-gel: The precursors are initially dissolved in a suitable solvent and colloidal particles are formed as a result of a set of chemical reactions. This colloidal suspension is known as sol (Figure 4b). These nanoscale sol particles are crosslinked and hierarchically assembled into an interconnected, porous network filled with liquid phase (Figure 4c). The resulting three-dimensional open grid structure is called the gel. Some gels such as polymeric gels can directly be formed from linear polymers instead of a precursor solution without the intermediate occurrence of individual particles.

(ii) Aging: The subsequent step is the aging of the tenuous solid skeleton of the alcogel generated during the sol-gel process. This process increases the connectivity of the alcogel network and its fractal dimension which reinforces the alcogel skeleton (Figure 4d).

(iii) Drying: The last step in the aerogel synthesis is the removal of the liquid solvent from the gel. Drying methods include ambient pressure drying (evaporation), freeze-drying, and supercritical drying. During drying at ambient pressure, the formation of a liquid–vapor phase boundary inside the pores results in a very high capillary pressure, which can cause the collapse of the pores and cracking of the gels. Cabot Corporation produces silica aerogels by ambient pressure drying; however, these aerogels are not monolithic but consist of irregularly shaped particles. Therefore, they would not be suitable for wave-guiding applications. In freeze-drying, the solvent temperature is reduced below its crystallization temperature and it is subsequently removed by sublimation at a reduced pressure. This technique is also problematic for the production of monoliths as the solvent crystallization may cause volume expansion and stress, which results in the breakage of the gels. Currently, freeze-drying is not used on a commercial scale to produce aerogels. Differential strain arising from capillary pressure can be eliminated by using the supercritical drying process. Supercritical drying of the alcogel is extraction of the solvent in its pores with supercritical fluids, commonly scCO_2_. During supercritical drying, the formation of two phases (a liquid and a vapor) in the pores is prevented, and, therefore, the collapse of the porous gel network can be avoided and a highly porous aerogel structure can be obtained [58,59] (Figure 4e).

### 4.2. Surface Modification of Aerogels

Some types of aerogels are hydrophilic due to the presence of polar hydroxyl groups within their porous framework. These hydroxyl groups can take part in strong hydrogen bonding with water, which can result in a structural collapse in a humid environment. However, the presence of non-polar hydrophobic groups on the aerogel surfaces renders them hydrophobic. Therefore, for optofluidic applications requiring aqueous cores, hydrophilic surface groups of aerogel monoliths need to be replaced by hydrophobic groups so that they can meet the stability requirements for long-term use [32,60]. For silica aerogels, this replacement of the surface groups can be achieved by three primary techniques. One approach is to utilize organosilanes other than TEOS that contain hydrophobic groups and intermediary chains; these hydrophobic organosilanes can be incorporated into the aerogel network as additional silica precursors in the synthesis. Examples of possible hydrophobic co-precursors include methyltrimethoxysilane (MTMS), methyltriethoxysilane (MTES), dimethylchlorosilane (DMCS), trimethylethoxysilane (TMES), ethyltriethoxysilane (ETES), and phenyltriethoxysilane (PTES). The silyl groups of the co-precursors react with the silica network during the condensation reactions, resulting in aerogels with different degrees of hydrophobicity [33,61]. Surface modification of aerogels can also be performed either during the aging step or after the drying step by vapor-phase deposition and supercritical-phase deposition techniques. For example, hydrophilic nanocellulose aerogel surfaces were transformed into hydrophobic ones by using hydrophobic molecules such as triethoxyl(octyl)silane and MTMS by either vapor phase deposition or liquid phase deposition techniques [62,63,64]. Similarly, the hydrophilic surface of a silica aerogel could be modified via a reaction with gaseous methanol for 10 h [65]. The treatment led to extremely hydrophobic aerogels that could even float on water for several hours without getting wet [66]. Hexamethyldisilazane (HMDS) can also be used as a surface modification agent either in pure vapor form or as a solution in scCO_2_ [33,67] Aerogels with contact angles as high as 130° could be obtained using HMDS dissolved in scCO_2_ [33,68]. The surface of the silica aerogels can also be modified by amine groups that can be incorporated from either the gas phase or the liquid phase [61]. To functionalize from the liquid phase, the alcogel can either be prepared with 3-(aminopropyl)-trimethoxysilane (APTMS) as the catalyst or it can be aged in APTMS containing an aging solution [33,61].

### 4.3. Channel Formation in Aerogel Monoliths

**Figure 5:**
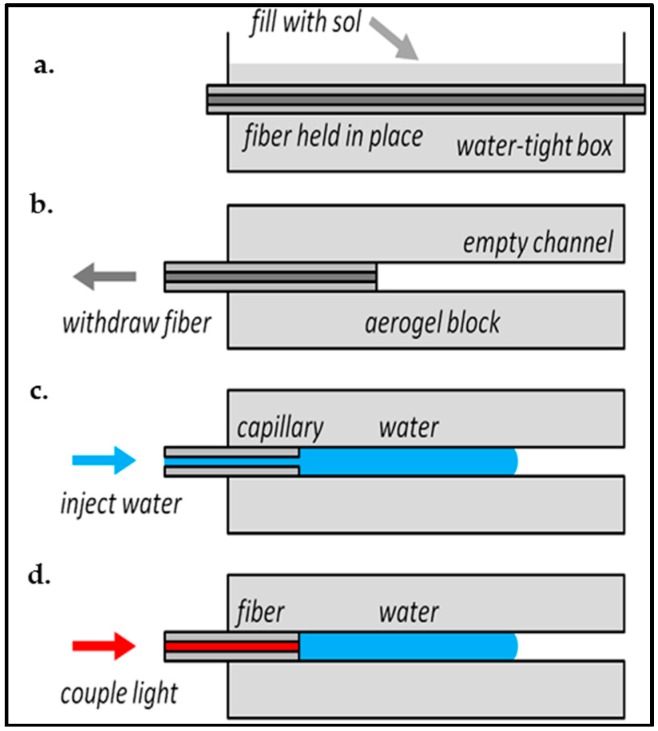
Formation of optofluidic waveguides in an aerogel monolith. (**a**) The precursor sol is poured over a fiber held in a box, allowed to gel, then processed into an aerogel; (**b**) the fiber is pulled out of the aerogel block to leave a microchannel; (**c**) water is pumped into the microchannel through a capillary; (**d**) light is coupled into the water column via a reinserted fiber [25]. “Reproduced with permission from Xiao et al., Optofluidic microchannels in aerogel; published by *Opt. Lett.*, 2011.”

There exist several possible strategies for fabricating uniform, extended channels in hydrophobic aerogel blocks [19,23,25,69]. Xiao et al. [25] demonstrated for the first time that microchannels in silica aerogel blocks can be used as optical waveguides. Figure 5 illustrates the steps for the formation of optofluidic waveguides in the aerogel. First, a solution of TEOS, water, and ethanol was poured into a mold in which an optical fiber with the cross section of the desired waveguide was placed; this fiber served as the channel preform (Figure 5a). After gelation of the solution, the resulting alcogel with the fiber inside was aged in methanol for at least two days. The alcogel with the embedded fiber was then soaked in 20 wt % HMDS solution in methanol for one day to replace most of the surface hydroxyl groups (–OH) with trimethylsilyl (–OSi(CH_3_)_3_), which rendered the aerogel surface hydrophobic. It was then immersed in pure methanol to remove unreacted HMDS from the aerogel structure [24,25,67]. Methanol within the alcogel was removed by supercritical extraction by scCO_2_. As shown in Figure 5b, the fiber was carefully pulled out of the aerogel block, leaving a fiber-sized microchannel within the block.

In addition to a single, straight microchannel, two parallel microchannels as well as Y-junction open channels could be created in the aerogel blocks following the steps in Figure 5a,b using a twisted pair of fibers. However, withdrawal of the fibers embedded in the aerogel frequently leads to breakage due to the adhesion of the fiber to the silica aerogel network. Furthermore, this technique only allows for limited channel shaping and sizing capabilities.

**Figure 6:**
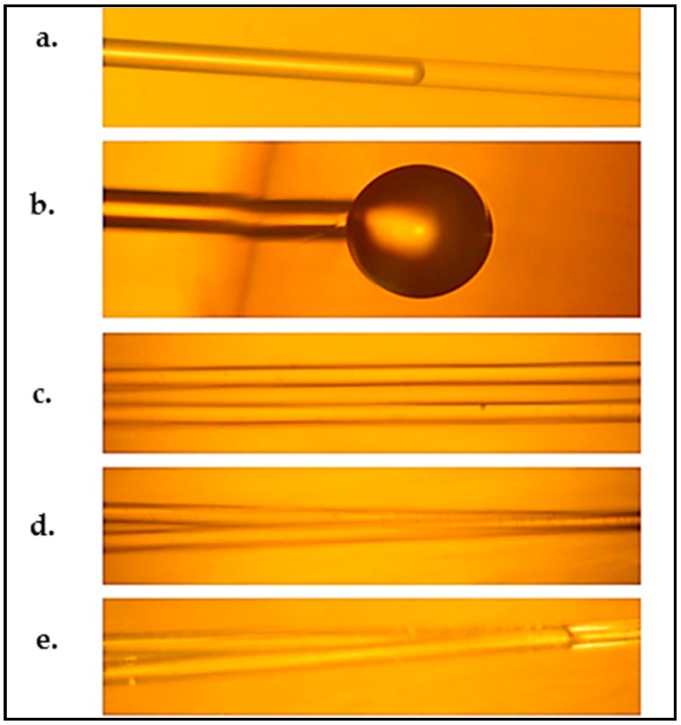
Water in 125 μm diameter microchannels in aerogel. (**a**) A water column (injected from the left) partly filling a microchannel; (**b**) the spherical droplet at the end of a completely filled microchannel; (**c**) two parallel air-filled microchannels; (**d**) combining at a Y-junction; (**e**) two separate water columns merging at the Y-junction shown in (**d**) [25]. “Reproduced with permission from Xiao et al., Optofluidic microchannels in aerogel; published by *Opt. Lett.*, 2011.”

Since the resulting aerogel was hydrophobic, the microchannels could not be filled with water by simple immersion in water and capillary forces. Thus, they had to be filled by a syringe connected to a fiber-sized capillary that was carefully inserted into one end of the channel, as shown in Figure 5c and Figure 6. Air bubbles formed during the filling of the channel were removed by simply pushing the water column or the fiber towards the other end; the air in between quickly escaped sideways through the pores, leaving a continuous path without any bubbles.

**Figure 7:**
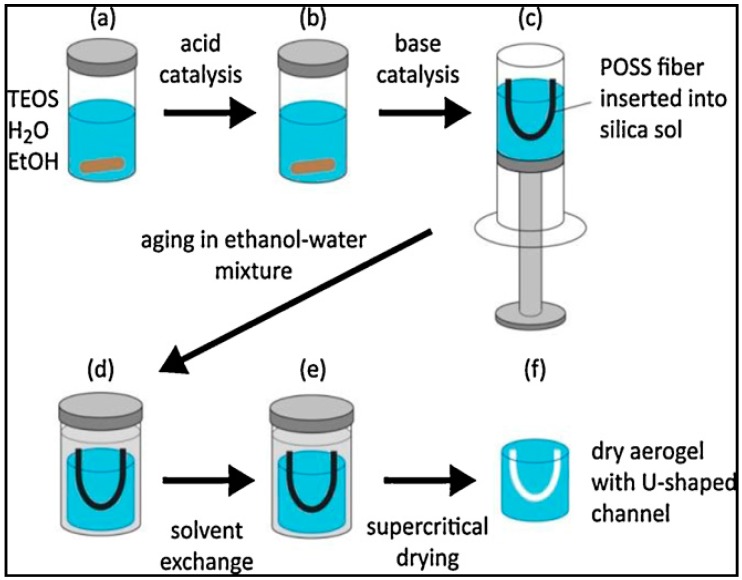
Preparation of silica aerogel monoliths with an embedded U-shaped channel [23]. (**a**) Precursors are mixed in the reaction vessel; (**b**) acid and (**c**) base catalysis lead to the formation of sol into which the channel preform made of POSS is inserted; (**d**) aging in ethanol-water mixture and (**e**) solvent exchange for pure ethanol yield reinforced alcogel; (**f**) extraction with scCO_2_ removes the POSS channel preform, leaving the final monolithic aerogel with an empty channel inside. “Reproduced with permission from Eris et al., Three-dimensional optofluidic waveguides in hydrophobic silica aerogels via supercritical fluid processing; published by *J. Supercrit. Fluids*, 2013.”

Eris et al. [23] formed channels in aerogels by using channel preforms made of trifluoropropyl Polyhedral Oligomeric Silsesquioxane (POSS), which is highly soluble in scCO_2_ and insoluble in the ethanol–water mixture used in the solvent exchange step. In order to prepare the channel preform, trifluoropropyl POSS was initially melted and then formed into a U-shaped fiber during solidification at room temperature. The solution of TEOS, water, ethanol, and acid catalyst was mixed and a base solution was added to the solution as shown in Figure 7a,b. The U-shaped trifluoropropyl POSS fiber was then placed in the solution, as seen in Figure 7c. The curved part of the fiber was kept inside the solution and its ends protruded from the upper surface. After gelation and aging of the alcogel with embedded POSS fiber in ethanol (Figure 7d,e), scCO_2_ extraction at 313.2 K and 9 MPa was performed to extract ethanol and the POSS fiber from the alcogel network. The extraction yielded a U-shaped empty channel inside, as displayed in Figure 7f. The silica aerogel with an empty U-shaped channel was subsequently treated with HMDS in a high-pressure vessel at 333.2 K and 10.34 MPa to make it hydrophobic. The aerogel was immersed in a solution of HMDS in CO_2_ for 2 h. Subsequently, unreacted HMDS as well as the reaction products of HMDS with surface hydroxyl groups were extracted by scCO_2_. Extraction resulted in monolithic and hydrophobic silica aerogel with an empty channel inside. Using this technique, breaking of the aerogel can be prevented and it is also possible to form complex-shaped channels in the aerogel blocks. However, there are a limited number of polymers that are soluble in scCO_2_ and insoluble in the solvents or solvent mixtures used in the solvent exchange step, which limits the use of this technique.

**Figure 8:**
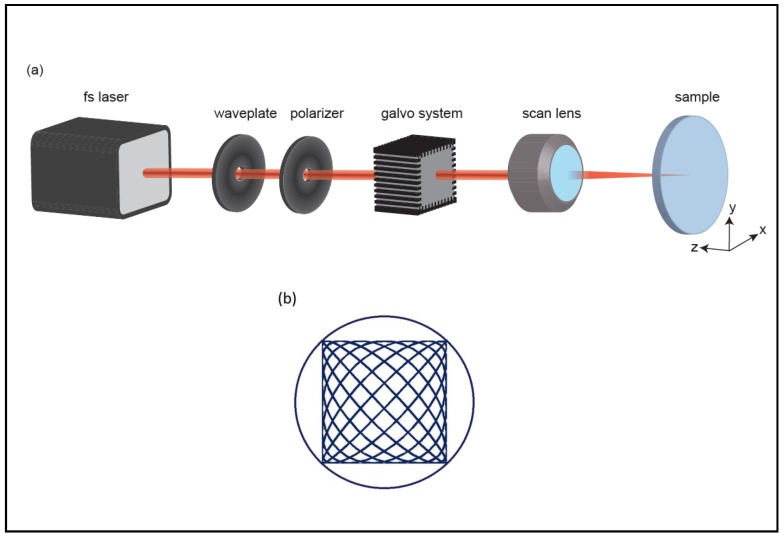
(**a**) Experimental setup used for aerogel micromachining by fs-laser ablation; (**b**) detail of the *xy*-scanning pattern of the ablation beam focus over the aerogel surface [19]. “Reproduced with permission from Yalızay et al., Versatile liquid-core optofluidic waveguides fabricated in hydrophobic silica aerogels by femtosecond-laser ablation; published by *Opt. Mater.*, 2015.”

Pilot studies of ultrafast-light micromachining of aerogels demonstrated that they can be precisely ablated with femtosecond lasers, thanks to their ultra-low thermal conductivity [69,70]. Sun et al. [69] demonstrated for the first time that silica aerogels can be machined through ultrafast laser processing. Bian et al. [70] also sliced cylindrical polyurea aerogel samples of 10–15 mm in diameter by laser ablation into 1–3 mm disks using femtosecond laser pulses. The laser beam at 800 nm was focused at the center of the sample, which was placed on a micro-positioning stage so that the sample could be rotated or translated in 3D for cutting. Following these initial demonstrations, Yalızay et al. [19] used femtosecond laser ablation to create channels in a hydrophobic monolithic silica aerogel with 0.2 g/cm^3^ density and refractive index of 1.037. Figure 8 shows the experimental setup used for micromachining channels with desired cross sections in aerogel blocks by laser ablation. The output power of the laser generating femtosecond pulses was adjusted by a combination of a polarizer and a half-wave plate. The direction of the laser beam propagation was controlled by a dual-axis scanning galvo system. The beam was focused on the aerogel surface by a scan lens and essentially vaporized the silica. Microchannels with desired cross sections were machined by sending sinusoidal driving signals of different frequencies to both *x*- and *y*-axis of the galvo scanner, resulting in the motion of the ablation beam focus along a Lissajous pattern (Figure 8b). An extended cylindrical channel inside the silica aerogel block was created by moving the aerogel along the *z*-axis of the system during the ablations process. Thus, cylindrical microchannels inside the silica aerogel blocks with length of ~5 mm and ~500–600 μm in diameter could be formed.

After the formation of microchannels inside aerogel monoliths, the channels were filled with ethylene glycol instead of water, using direct injection from a syringe. Since ethylene glycol is much less volatile than water, this permitted prolonged studies of the wave-guiding characteristics of open-ended channels.

**Figure 9:**
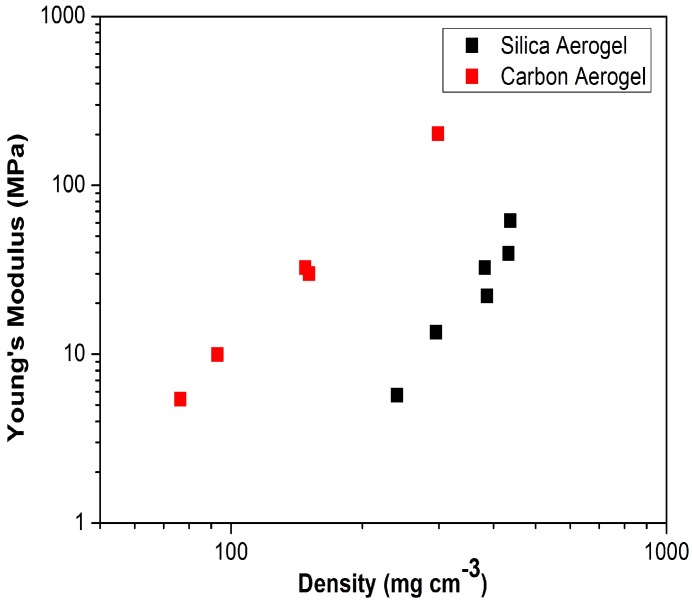
Dependence of Young’s modulus on density for carbon and silica aerogels [76]. “Reproduced with permission from Worsley et al., Mechanically robust and electrically conductive carbon nanotube foams; published by *Appl. Phys. Lett.*, 2009.”

As a possible alternative, long channels for liquid-core optofluidic waveguides of various shapes such as straight and inclined L-shape can also be manufactured in aerogels by a manual drilling technique. Silica aerogels can have a bulk density as low as 0.003 g/cm^3^, which is only 2.5 times higher than the density of air at 20 °C and 1 atm (0.0012 g/cm^3^). Such a low density translates into a high porosity of 99.9% and a very low refractive index of ~1.003, which makes such aerogels suitable for use as a cladding material [71,72,73]. However, the mechanical strength of such low-density aerogels is not too high since the mechanical strength of aerogels, like all porous materials, is strongly dependent on the load-bearing fraction of the solid in the aerogel network. Therefore, it is not practical to construct optofluidic devices from aerogels with such ultra-low densities. Figure 9 shows that Young’s modulus depends linearly on the density of monolithic carbon and silica aerogels [74,75,76]. Consequently, increasing the bulk density of the aerogel (decreasing the pore volume) will usually improve their mechanical strength. Aerogels with higher bulk densities and improved mechanical strength can be produced by using higher monomer content, by a drying process that collapses porosity, or by sintering [38,76,77].

**Figure 10:**
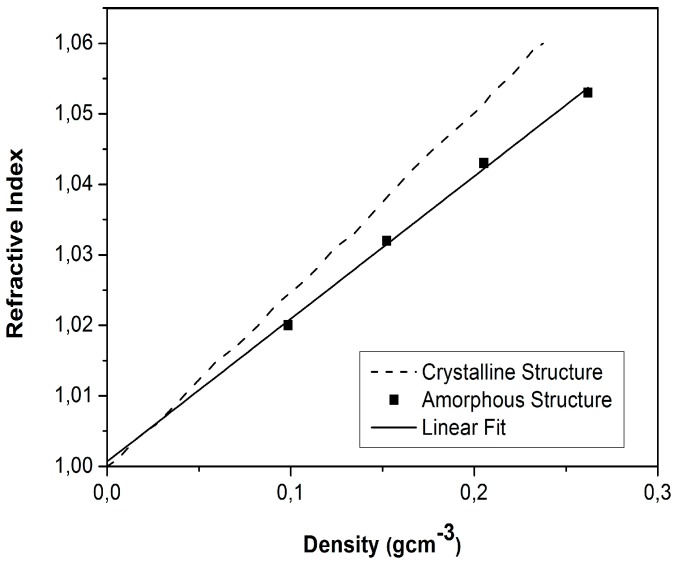
Refractive index as a function of silica aerogel density. The solid line assumes an amorphous structure (proportionality constant 0.21), while the dashed line assumes a crystalline structure [72]. “Reproduced with permission from Henning et al., Production of silica aerogel; published by *Physica Scripta*, 1981.”

According to the correlation described by Equation (1) and proposed by Henning et al. [72], which was also experimentally verified for aerogel samples with a density up to 0.3 g/cm^3^ (Figure 10), the refractive index (*n*) of aerogels increases linearly with their density, (ρ, g/cm^3^) [43,72,78,79]:

*n* = 1 + 0.21 ρ (1)

According to Equation (1), *n* is very close to 1 even for aerogels with higher densities. Thus, it is practical to use higher-density aerogels in optofluidics applications, as they provide increased mechanical stability without a significant deterioration of their optical characteristics.

An aerogel with higher density is still highly porous. When the light strikes the surface of channels fabricated in these aerogels, a large fraction of the channel wall is still formed by air and a fair amount of light can be totally internally reflected from this liquid–air interface (see Figure 2a). However, the light incident on the side walls of the channel in the aerogel also interacts with skeleton-forming primary and secondary particles (see Figure 2a,b); the secondary particles around 50 nm in diameter, surrounded by 100 nm pores, can then act as scattering centers and scatter the visible light in a wavelength-dependent manner. Thus, the increase in the bulk density and the solid content in the aerogel network may contribute to increased light losses due to the light absorption and scattering. In addition, increased wall surface irregularities created due to fabrication techniques also contribute to the scattering. The light striking such a rough surface bounces off in all directions due to multiple reflections by the irregularities [24,57,80,81,82], thus increasing the overall losses of the waveguide.

## 5. Characterization of Aerogel-Based Waveguides

**Figure 11:**
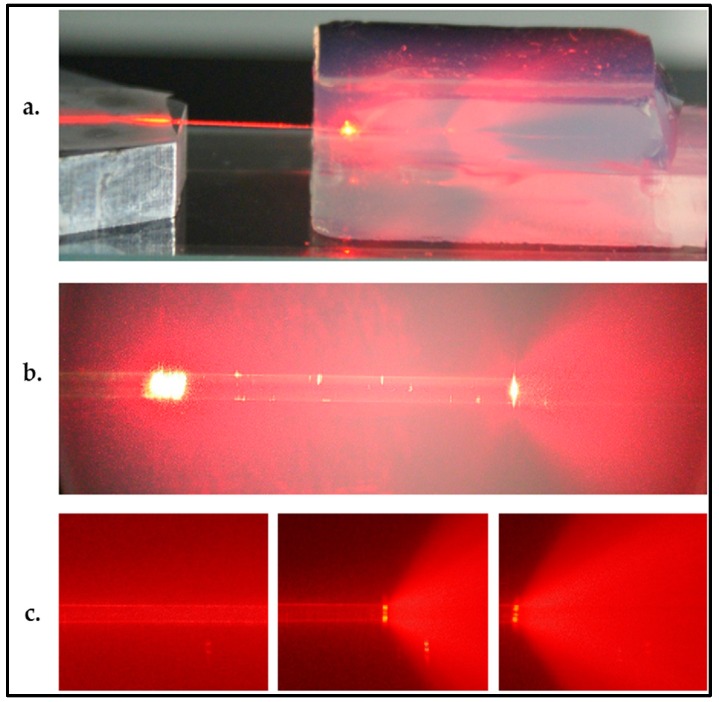
(**a**,**b**) Guiding of 635 nm laser light in water columns of different lengths in a 125 μm diameter aerogel microchannel. The light enters into the channel from the left through a fiber, and emerges from the end of the water column toward the right. The fiber ends at the bright scatter point about 2 mm inside the 10 mm long aerogel block; (**c**) (left to right) images taken at 40 s intervals at the end of a water column, as the water evaporates [25]. “Reproduced with permission from Xiao et al., Optofluidic microchannels in aerogel; published by *Opt. Lett.*, 2011.”

Xiao et al. [25] characterized the wave-guiding properties of water-filled channels created within aerogel monoliths, 125 μm in diameter, by coupling light into the channel via an optical fiber and measuring optical attenuation in the channel (see Figure 5d for illustration). A laser light with a wavelength of 635 nm was coupled into the other end of the fiber used in the characterization. Figure 11a,b show water-filled channels with light coupled into them. In both cases, diverging light beams emerge from the right end of the water-filled channel. Due to evaporation through the aerogel pores and the open channel end, the water inside the channel receded from right to left in a short span of time, resulting in the channel partially filled with air (Figure 11c). After emerging from the water-filled part of the channel, light could no longer be guided and its propagation was observed as a diverging cone of light due to light scattering from the aerogel particles (Figure 11b,c). 

As shown in Figure 11, laser light could be guided through the straight water-filled channel. The total waveguide loss including input and output coupling losses in the 16 mm long water-filled channel was measured as 2.4 dB. This loss corresponds to a waveguide attenuation of −1.5 dB/cm. As the authors pointed out, improved microchannel surface quality with low channel wall roughness owing to their technique may eliminate the losses due to Rayleigh scattering. In such a straight waveguide, the dominant loss mechanism should mostly be the propagation loss by the wavelength-dependent light absorption of the structure itself.

**Figure 12:**
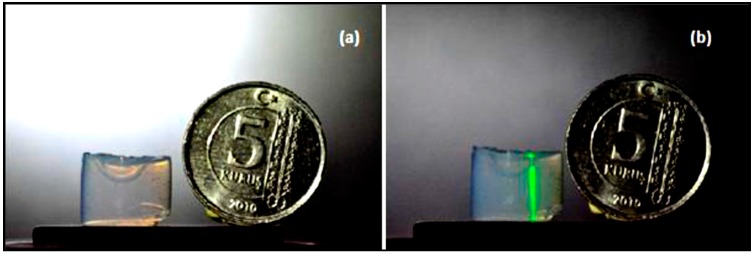
(**a**) Aerogel block with an empty U-shaped channel and (**b**) laser light focused from the top on one end of the channel before it was filled with water. Coin diameter is 17 mm [23]. “Reproduced with permission from Eris et al., Three-dimensional optofluidic waveguides in hydrophobic silica aerogels via supercritical fluid processing; published by *J. Supercrit. Fluids*, 2013.”

Eris et al. [23] also characterized the optofluidic waveguide efficiencies of their U-shaped channels (Figure 12a). A laser beam from a laser diode with a wavelength of 532 nm and 35 mW maximal output power was focused through a lens with a focal length of 150 mm at one open end of the channel in the aerogel placed on a two-dimensional translation stage. The position of the beam focus for maximal light transmission from the other end of the channel cross section was adjusted using the translation stage and visually monitoring of the intensity of light transmitted through the channel.

**Figure 13:**
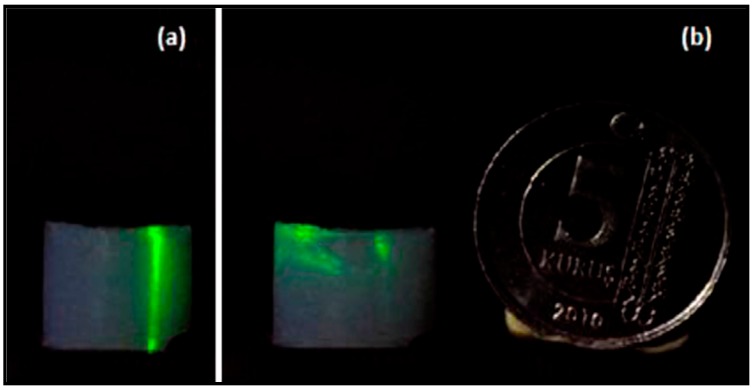
(**a**) Laser light focused from the top on the aerogel block outside of a water-filled channel and (**b**) laser light focused from the top on the right end of the water-filled channel. Coin diameter is 17 mm [23]. “Reproduced with permission from Eris et al., Three-dimensional optofluidic waveguides in hydrophobic silica aerogels via supercritical fluid processing; published by *J. Supercrit. Fluids*, 2013.”

**Figure 14:**
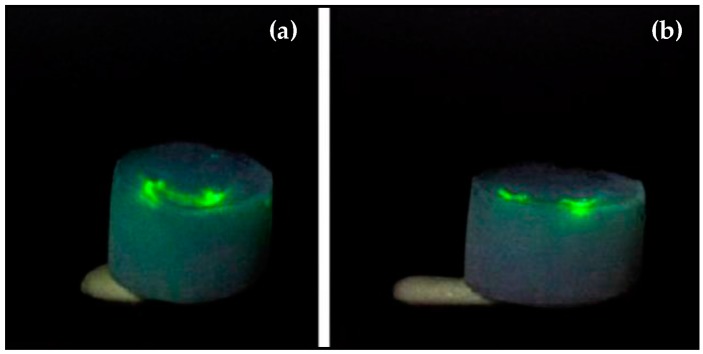
(**a**) Light guiding in a water-filled channel inside aerogel; (**b**) the light was focused on the left end of the channel and exited from the right end of the channel [23]. “Reproduced with permission from Eris et al., Three-dimensional optofluidic waveguides in hydrophobic silica aerogels via supercritical fluid processing; published by *J. Supercrit. Fluids*, 2013.”

The green laser beam was initially directed into the right end of an empty channel. Since the channel was filled with air, the laser light was transmitted straight through the aerogel without wave-guiding (Figure 12b). The channel was then filled with water and the light was directed into its right end again. The laser light coupled into the liquid core could be guided along the liquid-core optofluidic waveguide before exiting from the left, open end of the channel (Figure 13b). Figure 14 shows an additional example of light guiding in the water-filled channel; this time, the light enters the left end of the channel and emerges from the right end. Due to the U-shaped geometry of the liquid channel and the high divergence angle of the light transmitted through the channel, the fraction of the transmitted power could not be directly measured at the open end of the channel. However, a comparison of the images shown in Figure 13 and Figure 14 indicates that the intensities of light scattered from the input and output ends of water-filled channels are roughly similar; hence, it can be deduced that there is no substantial loss due to the scattering from the channel walls along the guided light path. For U-shaped waveguides, most of the loss is due to the bends in the waveguide, in which a fair amount of the energy of the guided mode is located close to the outer rim of the waveguide; therefore, the light can partially leak into the surrounding cladding. However, for aerogel-based waveguides, the high refractive index contrast between the core liquid and aerogel cladding enhances the light confinement in the waveguide, leading to significantly smaller bending losses. 

**Figure 15:**
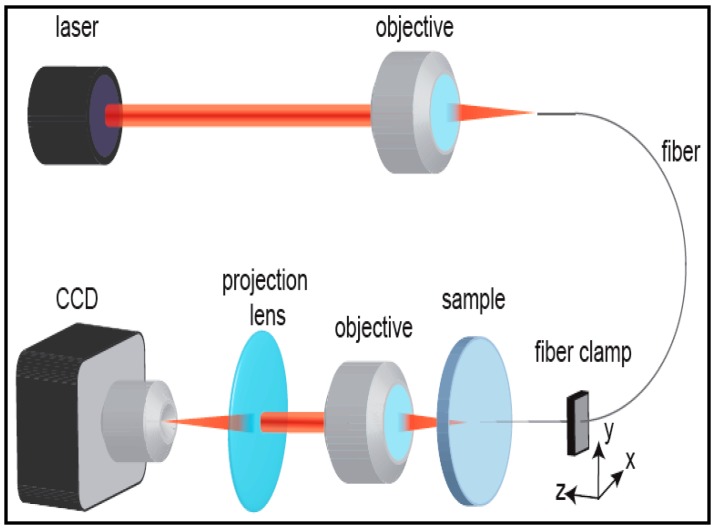
Schematics of experimental setup used in the characterization of the light-guiding performance of liquid core optofluidic waveguides [19,23]. “Reproduced with permission from Yalızay et al., Versatile liquid-core optofluidic waveguides fabricated in hydrophobic silica aerogels by femtosecond-laser ablation; published by *Opt. Mater.*, 2015.”

Yalızay et al. [19] used the experimental setup shown in Figure 15 for the characterization of light-guiding properties of their optofluidic waveguides and measurement of their propagation losses. An auxiliary laser at 632.8 nm was coupled into the liquid core of the waveguide through a tapered optical fiber ~5 μm in diameter and with a high NA of 0.57. The exit face of the liquid-filled channel output was imaged on a charge-coupled device (CCD) camera through a projection lens to quantify the amount of light transmitted through the waveguide.

**Figure 16:**
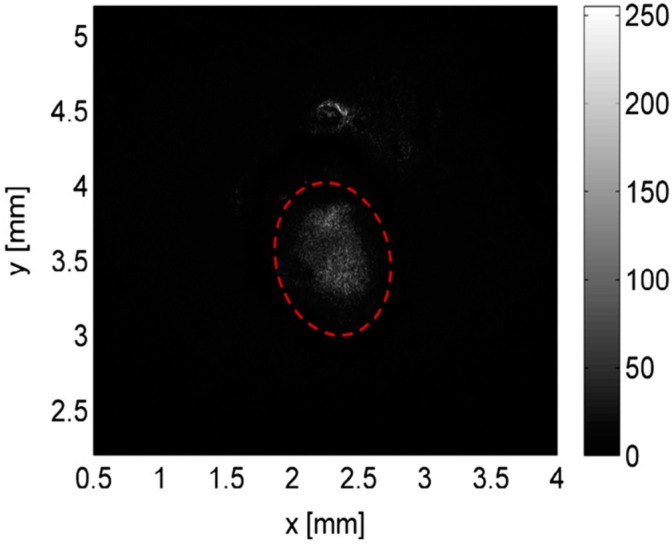
Grayscale charge-coupled device (CCD) camera image of guided light in the liquid-core waveguide embedded inside the aerogel. The red dashed curve indicates the channel contour, and the light outside the region delimited by the curve results from scattering losses [19]. “Reproduced with permission from Yalızay et al., Versatile liquid-core optofluidic waveguides fabricated in hydrophobic silica aerogels by femtosecond-laser ablation; published by *Opt. Mater.*, 2015.”

Subsequently, the tapered input fiber was moved along the channel axis and the total power of the light exiting the channel was simultaneously monitored as a function of the distance between the fiber tip and the channel output face. The grayscale values of all pixels in the obtained image of the waveguide exit face were added up to provide information on the transmitted light intensity. The images of the output intensity distribution were recorded for 100 μm increments of the input fiber position, moving forward along the channel axis. An illustration of a typical recorded image of the guided laser light is given in Figure 16. The recorded light distribution covering the entire channel cross-section contained within the red dashed curve shows that the coupled light was confined and guided inside the channel. The speckle pattern of the output light distribution in the image was attributed to the multimode behavior of the large-core waveguide and interference of individual guided modes dependent on the relative differences of their optical path length.

**Figure 17:**
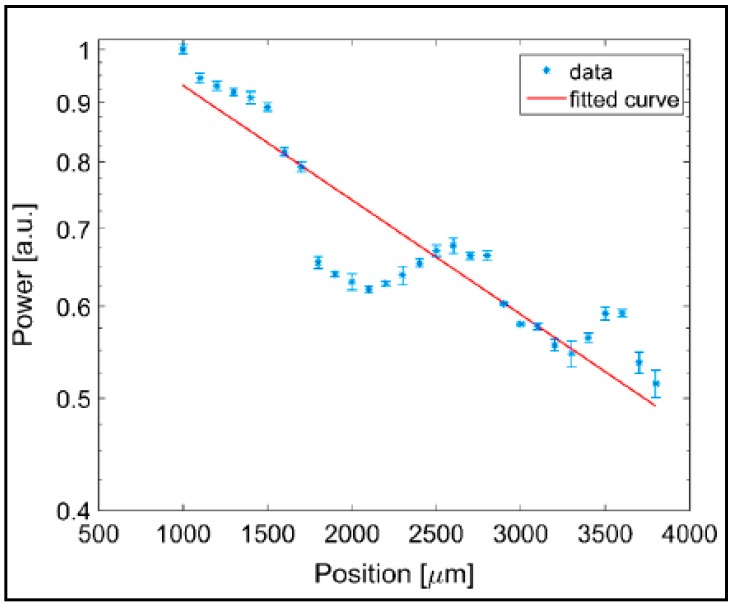
Normalized power of light transmitted through the waveguide versus the propagation distance along the waveguide axis, shown in semi-log scale. Blue dots: experimental points; red line: exponential fit of the experimental data [19]. “Reproduced with permission from Yalızay et al., Versatile liquid-core optofluidic waveguides fabricated in hydrophobic silica aerogels by femtosecond-laser ablation; published by *Opt. Mater.*, 2015.”

The total transmitted power integrated over the cross section of the channel was then plotted as a function of the propagation distance along the waveguide, as shown in Figure 17. The output power of the waveguide exhibited regular oscillations superimposed on an exponentially decreasing background. As argued above, these oscillations resulted from the path-dependent interference of multiple guided modes. The measured power of the transmitted light was fitted with an exponential curve as *P*(*z*) = *P*_o_exp(−α*z*) where *z* is the propagation distance along the waveguide axis and α is the attenuation coefficient. From the particular measurement shown in Figure 17, α was obtained as 2.278 cm^−1^. The propagation loss of the optical waveguide (η) was then calculated as −9.9 dB/cm using the equation η = −10α/ln10. This propagation loss is around 7 times higher than −1.5 dB/cm propagation loss obtained in the previous demonstration by Xiao et al. [25]. This difference is mainly due to increased roughness of the channel surfaces produced by laser micromachining, leading to higher scattering losses in the waveguides.

## 6. Applications of Aerogel-Based Optofluidic Waveguides

**Figure 18:**
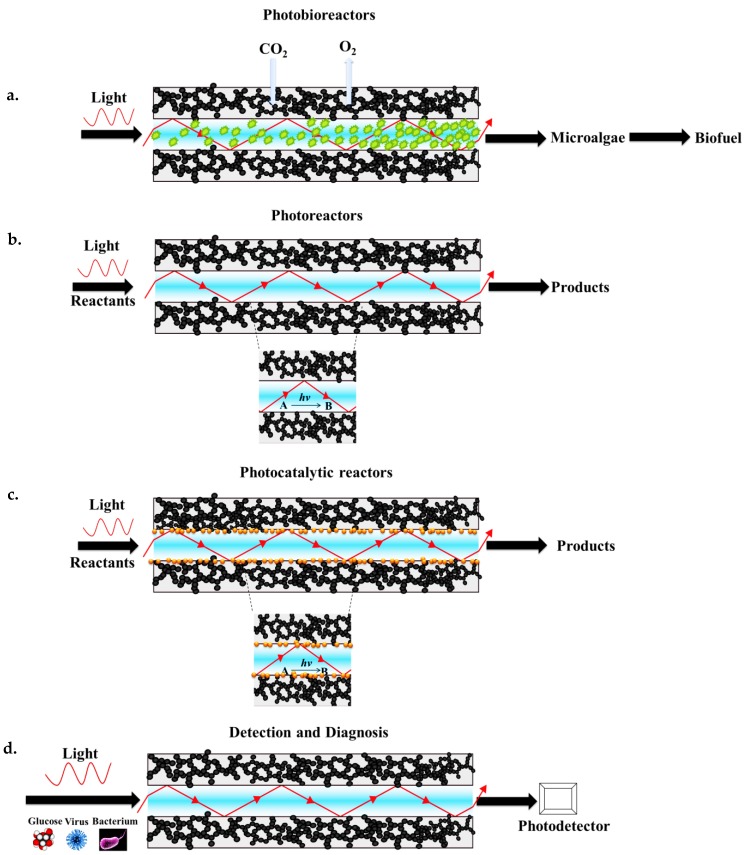
Possible applications of aerogel-based optofluidic waveguides. (**a**) Aerogel-based photobioreactors. (**b**) Aerogel-based photoreactors. (**c**) Deposition of suitable catalyst particles within the aerogel channel walls can improve efficiency of light-induced chemical reactions. (**d**) Detection and diagnostic microsystems based on optofluidic waveguides.

As summarized in Figure 18, aerogel-based optofluidic waveguides are promising devices for several applications including conversion of light energy in photochemical reactions carried out in photoreactors as well as light-assisted detection, identification, and quantification of particular chemical compounds, metabolic variables, and biological particles such as blood oxygen, glucose level, or viruses and bacteria suspended in the core liquid. Aerogel-based optofluidic waveguides can potentially also complement conventional photobioreactors (PBRs) providing enhanced light distribution to the organisms farther away from the illumination source and in confined places (Figure 18a). Since the porous aerogels readily allow the gases to be transferred between the microchannel and the surroundings, the CO_2_ required for biomass growth can diffuse into the channel and the product of photosynthesis, O_2_, can diffuse out through its pores (see also Figure 2a,b). 

In addition, aerogel-based photoreactors hold great promise for photochemistry. For instance, light-initiated synthesis of novel molecular building blocks and various types of polymerization reactions, as well as treatment of wastewater, including removal of highly complex organic compounds such as dyes, surfactants, and pesticides, to obtain high-purity water can be carried out in such reactors (Figure 18b). Photocatalytic reactions are another interesting area for the potential application of aerogel-based optofluidic waveguides. Their high porosity and high surface area with interconnected open pore structure, combined with a fully tunable three-dimensional structure, make aerogels effective for photocatalyst deposition in their structure, thus potentially improving photocatalytic activity. As shown in Figure 18c, microchannel walls in an aerogel monolith are porous and a photocatalyst can be immobilized within these pores with good adhesion to the solid network. The light can be partially absorbed in the near-surface region of the photocatalysts immobilized in the pores of the wall. Thus, the reactants can possibly be converted into desired products on the catalyst surface, effectively driving the reactions. Diagnostic monitoring of glucose and alcohol concentrations in the blood based on quantitative optical absorption measurements can possibly be carried out by the aid of efficient light-guiding properties of the aerogel clad-TIR waveguides. This can be performed by the injection of a blood sample to the waveguide, or by using the aerogel-based optofluidic chips as implantable light-delivery lab-on-a-chip devices, particularly for photomedicine (Figure 18d). Additionally, various foodborne pathogens such as bacteria, viruses, and pesticides can be detected based on optical absorption measurements.

## 7. Conclusions

Aerogels have a very low refractive index and absorption coefficient; these optical properties make them ideally suited to function as the solid cladding material of liquid-core optofluidic waveguides that can guide light by total internal reflection over a wide range of wavelengths, without the use of any additional optical coatings. Aerogel-based optofluidic waveguides can provide high numerical apertures in applications involving aqueous liquid cores, giving them a significant advantage over polymer-based materials. Typically, waveguides are constructed by opening channels inside monolithic aerogel blocks. Using processes such as preform removal or femtosecond laser ablation, uniform and extended channels in aerogel monoliths can be created. Channel preforms made of soluble polymers that can be removed from the aerogel during the scCO_2_ drying step of the aerogel synthesis can bring large flexibility to the production of structures with an arbitrary three-dimensional geometry. Alternatively, ultrafast micromachining of aerogels by femtosecond laser ablation might also enable fabrication of arbitrary three-dimensional paths within the aerogel monoliths. Followed by a surface-functionalization step, the channels in aerogels can be made compatible with both polar and non-polar core liquids. Since the porous aerogels readily allow gases to pass between the microchannel and the surroundings, air bubbles are not trapped in the channel and can diffuse out through the pores, which is favorable for effective light propagation and channel filling. At the same time, gases from the outside can pass through the channel walls and serve as reaction components for photochemical reactions carried out within the channels or as nutrients for photosynthetic organism cultivated inside the channels. Pilot studies presented in the literature to date revealed the potential of aerogel-based waveguides for efficient light routing in optofluidic light-wave circuits. Experimentally obtained propagation losses in aerogel-based waveguides were found to be comparable to the propagation losses measured previously for alternative liquid-core optofluidic waveguide platforms [83,84,85]. In particular, optical attenuation in the fabricated aerogel channels varied due to different surface scattering losses from a minimum of −1.5 dB/cm to approximately −10 dB/cm. Thus, aerogel-based optofluidic waveguides could pave the way for several innovative applications including photochemical reactions as well as light-driven detection and the identification and quantification of particular chemical compounds and metabolic variables with the aid of improved light distribution through the channel, even in confined geometries. The studies of aerogel-based optofluidic waveguides published so far have concentrated on the use of silica aerogels. However, other types of aerogels with interesting optical and mechanical properties (such as tensile strength or flexibility) can be adopted to enlarge the spectrum of possible applications. In addition, increasing the market availability of various aerogels makes these materials economically viable and can be promising for the production of various aerogels with different properties to be used in optofluidics-related applications on a commercial scale in the near future.
